# Changes in the Fungal Microbiome of Maize During Hermetic Storage in the United States and Kenya

**DOI:** 10.3389/fmicb.2018.02336

**Published:** 2018-10-02

**Authors:** Brett Lane, Sandeep Sharma, Chenxing Niu, Angeline W. Maina, John M. Wagacha, Burton H. Bluhm, Charles P. Woloshuk

**Affiliations:** ^1^Department of Botany and Plant Pathology, Purdue University, West Lafayette, IN, United States; ^2^Department of Plant Pathology, University of Arkansas, Fayetteville, AR, United States; ^3^School of Biological Sciences, University of Nairobi, Nairobi, Kenya

**Keywords:** metagenomic analysis, PICS, post-harvest, food security, aflatoxins

## Abstract

Prior to harvest, maize kernels are invaded by a diverse population of fungal organisms that comprise the microbiome of the grain mass. Poor post-harvest practices and improper drying can lead to the growth of mycotoxigenic storage fungi and deterioration of grain quality. Hermetic storage bags are a low-cost technology for the preservation of grain during storage, which has seen significant adoption in many regions of Sub-Saharan Africa. This study explored the use of high-throughput DNA sequencing of the fungal Internal Transcribed Spacer 2 (ITS2) region for characterization of the fungal microbiome before and after 3 months of storage in hermetic and non-hermetic (woven) bags in the United States and Kenya. Analysis of 1,377,221 and 3,633,944 ITS2 sequences from the United States and Kenya, respectively, resulted in 251 and 164 operational taxonomic units (OTUs). Taxonomic assignment of these OTUs revealed 63 and 34 fungal genera in the US and Kenya samples, respectively, many of which were not detected by traditional plating methods. The most abundant genus was *Fusarium*, which was identified in all samples. Storage fungi were detected in the grain mass prior to the storage experiments and increased in relative abundance within the woven bags. The results also indicate that storage location had no effect on the fungal microbiome of grain stored in the United States, while storage bag type led to significant changes in fungal composition. The fungal microbiome of the Kenya grain also underwent significant changes in composition during storage and fungal diversity increased during storage regardless of bag type. Our results indicated that extraction of DNA from ground kernels is sufficient for identifying the fungi associated with the maize. The results also indicated that bag type was the most important factor influencing changes in fungal microbiome during storage. The results also support the recommended use of hermetic storage for reducing food safety risks, especially from mycotoxigenic fungi.

## Introduction

Microbial communities interact with plant roots and above ground tissues, and can significantly impact plant health in positive or negative ways (Caporaso et al., 2010; Berendsen et al., 2012). A variety of factors influence the structure of the microbial communities, including plant genotype, management practices, and local environments (Doran, 1980; Aira et al., 2010). During the last decade, high-throughput sequencing (HTS) of DNA revolutionized the study of plant microbial communities by providing a more comprehensive picture of the community composition than traditional culturing techniques (Turner et al., 2013). The consensus among scientists is that a more detailed understanding of plant-associated fungal microbiomes could lead to new strategies for improved plant health and food safety (Singh and Trivedi, 2017).

Recent studies have utilized HTS to advance the current understanding of how storage conditions influence the fungal microbiome of various agricultural products. During storage of sugar beets, temperature had the most significant effect on the fungal microbiome composition (Liebe et al., 2016). At 8°C, *Botrytis cinerea* was the most prevalent species, while *Gibberella* and *Penicillium* species were predominant at 20°C. Additionally, species richness increased among the *Gibberella* during storage while other genera underwent little change. Studies on peanut identified only eight genera by traditional plating methods (Fernandez et al., 1997; Bhattacharya and Raha, 2002; Nakai et al., 2008). A study by Ding et al. (2015) explored the fungal microbiome of stored peanuts over 12 months of storage in four providences of China by HTS of the fungal ITS2 region. The authors identified between 22 and 43 genera in each location including *Aspergillus*, *Clonostachys*, *Emericella*, *Eurotium*, *Penicillium*, and *Rhizopus*. The presence of *Aspergillus* within the samples was noted to be higher in the samples from warmer Southern latitudes and increased in prevalence during storage. A further study by Xing et al. (2016) identified a total of 41 genera in whole peanuts and 37 genera in the shelled seeds. The authors also detected a shift in fungal composition over time during storage; the relative abundance of *Aspergillus* species increased with longer storage. A study by Chen et al. (2016) of the mycoflora of barley heads at harvest determined that geographic location was the primary factor affecting the microbiome composition of the grain.

In recent years, hermetic storage bags have been proven to be an effective technology for preserving grain quality during storage (Murdock and Baoua, 2014; Williams et al., 2014; Ng’ang’a et al., 2016). Grain placed in a hermetic bag experiences a different environment compared to grain in a traditional woven bag. Hermetic bags create a high carbon dioxide, low oxygen environment because the plastic material is impermeable to gasses and moisture (Murdock and Baoua, 2014). Multiple studies have demonstrated that quality of grain is maintained within the hermetic bags during storage (Murdock et al., 2012; Navarro et al., 2012; Baoua et al., 2014; Williams et al., 2014). In contrast, woven bags allow the exchange of oxygen, carbon dioxide and moisture, which leads to the rapid growth and development of insects and fungi. The effect of hermetic storage on the stored grain fungal microbiome remains unknown. The current study stems from two previous studies in the United States and Kenya that examine the efficacy of storing maize in hermetic bags to reduce the risk of mycotoxin accumulation during storage. In both studies, maize was sealed in hermetic and woven bags and stored for 3 months at three locations, Indiana and Arkansas, United States, and Makueni County, Kenya (Maina et al., 2016, 2017; Lane and Woloshuk, 2017). Maize stored in the United States was harvested from a single source, while in Kenya individual farmers grew, dried and stored the maize at their farms. The bags stored in Arkansas experienced much warmer conditions than those stored in Indiana. The temperature in Arkansas was over 30°C for 61 days compared to 1 day in Indiana (Lane and Woloshuk, 2017). Also, the night time lows in Indiana were below 20°C for 51 days compared to only 5 days at the Arkansas location. Relative humidity was also different at the two United States locations, with Indiana experiencing more days of high relative humidity (>85% RH) and less days with low humidity (<65% RH) (Lane and Woloshuk, 2017). Data temperature/humidity data-loggers were not available for the Kenya experiment, however, the temperature range was 20–29°C and the relative humidity was 44–96% (Maina et al., 2016). These previous studies demonstrated that hermetic storage bags prevent growth of mycotoxigenic storage-fungi and the accumulation of mycotoxin in the grain (Maina et al., 2016, 2017; Lane and Woloshuk, 2017). In this current study, our objective was to use HTS methods to compare the fungal microbiomes of the maize stored in hermetic and woven bags at the different geographic locations.

## Materials and Methods

### Grain Samples

Maize grain was obtained from prior studies in the United States and Kenya that compared the efficacy of Purdue Improved Crop Storage (PICS) hermetic bags with woven polypropylene bags (Maina et al., 2016, 2017; Lane and Woloshuk, 2017). In these studies, samples were taken from throughout the grain mass prior to storage. The maize was then placed in 50 kg PICS and woven polypropylene bags. Air was displaced from the headspace of the bags and bag openings were tightly folded shut and sealed with zip ties. Bags were then stored under local environmental conditions for 3 months.

The maize stored in the United States was grown in Tippecanoe County, IN, United States and was obtained from a single source (Buck Creek Elevator, Buck Creek, IN, United States). The maize was stored by the producer through the winter prior to commencement of the storage experiment. The grain was graded United States number five due to the large amount of broken kernels, foreign material, and grain dust. Grain samples were transported to and stored at two locations, West Lafayette, Indiana and Marianna, Arkansas. Two zero-time samples and 12 storage samples, were obtained from two treatments (three PICS bags and three woven bags) at both locations, West Lafayette, Indiana and Marianna, Arkansas. From each United States sample (750 g), five subsamples of 30 g were placed into 250-ml flasks containing 50 ml of 0.05% Triton X-100 and shaken for 1 min at 150 rpm in an environmental incubator shaker (New Brunswick, Scientific Co Inc., Edison, NJ, United States). Afterward, the wash was centrifuged at 1750 × *g* in a clinical centrifuge for 10 min and the pellet was suspended in 2 ml of 0.05% Triton X-100. Maize (150 g) from each United States sample also was ground in a Fresh Grind Coffee Grinder (Hamilton Beach, Glen Allen, VA, United States) and thoroughly mixed. The maize stored in Kenya was grown by 30 local farmers in Kaiti Division, Makueni County and was stored immediately after harvest and drying. Each farmer had a single PICS bag (50 kg) and woven bag. Representative maize grain samples (500 g) were collected from throughout the storage bags before and after 3 months of storage and sent to Purdue University. The entire sample was ground with the coffee grinder and thoroughly mixed.

### DNA Extraction

For DNA extraction, 1.5 ml of the kernel-wash suspensions were transferred to 2-ml screw-cap microcentrifuge tubes containing 0.5 mm glass beads (Scientific Industries Inc., Bohemia, NY, United States). For all ground samples, three sub-samples of 0.3 g were placed in 2-ml screw-cap microcentrifuge tubes containing about 1.25 g of glass beads and CTAB extraction buffer (Saghai-Maroof et al., 1984). Samples were homogenized with a Mini-Beadbeater system (Biospec Products, Bartlesville, OK, United States) by three 1-min shakes on the bead beater at 4800 rpm with 1 min on ice between each shake. The tubes were then incubated for 15 min at 65°C and centrifuged at 16,000 × *g* for 5 min. The supernatant (500 μl) was transferred to a fresh microcentrifuge tube, extracted with phenol:chloroform, and the DNA precipitated with ethanol (Raeder and Broda, 1985). For the kernel-washes, DNA from the five subsamples were then combined, as were the three subsamples from the ground kernels.

### Sequencing and Analyses

The ITS2 region of the fungal rDNA was amplified by PCR with modified ITS4 and fITS7 primers (White et al., 1990; Ihrmark et al., 2012; Toju et al., 2012). The ITS4_A_MID primer contained the ITS4 sequence, barcode nucleotides (represented by *x*) and the Adapter A sequence used in Ion Torrent sequencing (Thermofisher Scientific, Waltham, MA United States), 5′CCATCTCATCCCTGCGTGTCTCCGACTCAG*xxxxxxxxxx*G ATTCCTCCGCTTATTGATATGC3′. The fITS7-trP1 primer contained the fITS7 sequence and the truncated P1 adapter sequence, 5′CCTCTCTATGGGCAGTCGGTGATGTGAR TCATCGAATCTTTG3′. The PCR reaction mixture consisted of 0.5 μl of 10 mM of each primer, 0.5 μl of 10 mM dNTP mix, 5 μl of 5X Crimson *Taq* reaction buffer (New England Biolabs, Ipswich, MA, United States), 0.2 μl of Crimson *Taq* DNA polymerase (New England Biolabs, Ipswich, MA, United States) and 200 ng of template DNA in a total reaction volume of 25 μl. The amplification conditions were set at 30 s of initial denaturation at 95°C, followed by 30 cycles of 95°C for 30 s, 52°C for 30 s, and 68°C for 30 s, and final step of 68°C for 5 min. The PCR products were purified with equal volumes of Agencourt AMPure XP magnetic beads (Beckman Coulter, Brea, CA, United States) and quantified with a Quant-iT PicoGreen dsDNA Assay Kit (Thermofisher Scientific, Waltham, MA United States) on Nanodrop ND3300 fluorospectrometer (Thermofisher Scientific). The samples were pooled in equimolar amounts and the pooled DNA was diluted to 8 pM. DNA sequencing was conducted on an Ion Torrent Personal Genome Machine (Thermofisher Scientific, Waltham, MA United States) with 318 V2 Chip kit.

BAM-formatted data from the DNA sequencer were converted to fastq format with Bamtools (Barnett et al., 2011), and then to Sanger format (Cock et al., 2010). Sequencing reads smaller than 275 bp and larger than 500 bp were discarded (Blankenberg et al., 2010). The fastx toolkit was then used to trim reads to 275 bp from the 3′ end and filter sequences so at least 90% of the base pairs in each read had a Phred score of at least 20 (Gordon and Hannon, 2010, unpublished). All the above steps were accomplished on the Galaxy Server hosted by Penn State (Afgan et al., 2016). The output sequences from the United States and Kenya were combined into respective groups and clustered with the UCLUST algorithm within QIIME (version 1.9.1) (Caporaso et al., 2010; Edgar, 2010). Clusters containing ≥5 reads with 98% sequence similarity were defined as operational taxonomic units (OTU). A similarity index of 98% was used for the formation of OTUs to increase the number of clusters formed and increase differentiation (Kõljalg et al., 2013). The most abundant sequence from each OTU was selected as the representative sequence, and taxonomic assignment (genus) was made by BLAST analysis to the UNITE database (Kõljalg et al., 2013). The taxonomic assignment was based on a minimum identity of 97% and we selected the genus with the highest identity score. Based on available literature, genera were categorized as field fungi, storage fungi, yeast-like fungi, or other (unknown) fungi. Plant pathogenic fungi not previously reported on maize were included in the other category. As a final step, OTUs containing less than 0.01% of the total sequences were discarded.

### Statistical Analysis

QIIME software contains several statistical applications, including the analysis of alpha and beta diversity, which are computed using the R packages ape and vegan (Paradis et al., 2004; Caporaso et al., 2010; Kuczynski et al., 2011; Oksanen et al., 2011; Battaglia, 2017). Alpha and beta diversity values were computed with the workflow “core_diversity_analyses.py.” In order to minimize the effect of unequal sequencing, all rarefaction was preformed to the depth of the smallest sample in a given analysis. Alpha diversity indices are a measure of diversity within individual sites or treatments and can also be used to compare the overall diversity of sites or treatments. In this study, alpha diversity measures were used for a comparison of OTU diversity between replicates of each treatment at each storage site. The software calculated the intra-sample alpha diversity distances with the Chao1 index (Chao, 1984), the observed species, and the phylogenetic distance (PD) whole tree metric (Faith, 1992) using the default settings. Alpha diversity measures were compared between samples with non-parametric Monte Carlo simulations using 999 permutations (Metropolis et al., 1953). Multiple comparisons were corrected with a Bonferroni protocol (Bonferroni, 1935). Beta diversity indices measure the dissimilarity of sample composition between sites or treatments and are used to compare microbial composition between populations. In this study, beta diversity indices were used to compare OTU composition and read abundance between treatments and storage sites. Inter-sample beta diversity was calculated with phylogenetic (weighted and unweighted UniFrac) (Lozupone and Knight, 2005) and the non-phylogenetic (Bray-Curtis dissimilarity) metrics (Bray and Curtis, 1957). Comparisons of beta diversity measures were made using the workflow “compare_categories.py” using a non-parametric analysis of similarities (ANOSIM) with 999 permutations (Anderson and Walsh, 2013). To determine significant changes in the relative abundance of individual OTUs after 3-months of storage, a pairwise non-parametric *t*-test using 1000 Monte Carlo simulations was applied to the data through the QIIME workflow “group_significance.py” (Shaw et al., 2014; Livanos et al., 2016). Multiple comparisons of the relative abundance of OTUs were performed using a Kruskal–Wallis one-way analysis of variance and corrected with the Bonferroni protocol (Kruskal and Wallis, 1952).

## Results

### Assignment of Operational Taxonomic Units (OTUs)

The average number of raw DNA sequence data for each grain sample was about 100,000 reads. After filtering for quality and size, approximately 50% of the reads from each sample remained. A total of 811,049 quality reads were obtained from the ground maize samples in the US and 566,172 from the kernel-wash samples. Additionally, a total of 3,633,944 quality reads were obtained from the maize samples from Kenya. The average number of quality reads per sample were greater than 49,000 and 40,000 for the United States and Kenya samples, respectively. To estimate the coverage of sequencing we applied Good’s protocol to the data prior to the removal of the low abundant OTUs. The value was estimated to be greater than 99%, which indicates sufficiently deep coverage of the microbiome (Good, 1953; Hartmann et al., 2014).

From the United States samples, 213 total OTUs were identified from the kernel-washes and 136 total OTUs from the ground samples (**Table 1**). These OTUs contained 96.9% of the total quality reads. An average of 91% of the OTUs detected after storage were also identified in the initial grain, sampled prior to storage. We determined core members in the fungal microbiome, which describes members of the microbiome that are common between all samples (Turnbaugh et al., 2007). In our United States experiment, there were 107 OTUs in the core. When the OTUs were separated into DNA isolated from kernel-washes and ground kernels, the number of core OTUs was 125 and 110, respectively. A comparison of beta diversity in the kernel-washes and ground kernel samples revealed significant (*P* < 0.05) differences between the extraction methods. Because of these differences, kernel-wash and ground kernel data were separated in further analyses. Analysis of sequences from Kenya identified 164 OTUs, which comprised 95.6% of the total quality sequences. Each sample contained an average of 130 total OTUs (**Table 2**). On average, 84% of the OTUs identified in the initial grain samples were also found after 3 months of storage. With respect to storage bag type, about 90% of the OTUs were determined to be core to all treatments after 3 months.

**Table 1:** Number of OTUs identified from maize stored in Indiana (IN) and Arkansas (AR) in the United States after 3 months of storage.

	Initial	IN PICS	IN Woven	AR PICS	AR Woven	Core^a^
Ground	118	123 (93^b^)	130 (88)	126 (93)	133 (87)	110
Wash	177	192 (89)	178 (90)	182 (88)	167 (87)	125
Overall	166	173 (94)	171 (94)	170 (94)	176 (92)	107

^a^*Core OTUs contain sequences from all bag types. ^*b*^In parentheses: Percent OTUs in treatment-samples that were also identified in the initial grain sample prior to storage*.

**Table 2:** Number of OTUs identified from maize stored on farms in Kenya after 3 months of storage.

Sample	Initial	PICS	Woven	Core^a^
Farm 1	79	86 (57^b^)	107 (57)	33
Farm 2	81	122 (49)	39 (56)	13
Farm 3	117	103 (94)	99 (91)	71
Farm 4	70	103 (50)	108 (52)	38
Farm 5	50	77 (36)	109 (37)	16
Farm 6	71	90 (51)	122 (47)	31
Farm 7	73	100 (51)	97 (51)	34
Farm 8	130	100 (96)	110 (93)	74
Farm 9	54	104 (41)	56 (41)	16
Farm 10	60	88 (47)	106 (44)	31
Farm 11	79	93 (56)	87 (56)	35
Farm 12	67	112 (47)	104 (50)	38
Farm 13	70	101 (51)	110 (46)	37
Farm 14	85	118 (58)	114 (58)	52
Farm 15	70	102 (53)	100 (53)	39
Farm 16	70	107 (50)	118 (47)	38
Farm 17	73	100 (57)	109 (52)	42
Farm 18	75	101 (52)	126 (50)	41
Farm 19	70	108 (51)	112 (52)	40
Farm 20	80	106 (58)	121 (57)	52
Farm 21	69	92 (55)	105 (52)	40
Farm 22	59	72 (50)	82 (49)	27
Farm 23	65	72 (47)	116 (46)	23
Farm 24	77	94 (62)	131 (53)	45
Farm 25	81	110 (55)	114 (55)	49
Farm 26	66	87 (53)	98 (50)	33
Farm 27	75	101 (53)	104 (54)	40
Farm 28	63	85 (46)	86 (43)	23
Farm 29	68	93 (53)	106 (52)	38
Farm 30	70	111 (54)	90 (50)	31

^a^*Core OTUs contain sequences from all bag types. ^*b*^In parentheses: Percent OTUs in treatment-samples that were also identified in the initial grain sample prior to storage*.

### Taxonomic Assignments

Representative OTU sequences were used to query the UNITE fungal database using CONSTAX (Gdanetz et al., 2017), a pipeline which determines taxonomic assignment through a consensus of UTAX, SINTAX, and the RDP classifier. From the analysis, a total of 63 genera were identified in the United States maize. Of these, 42 were present in the ground samples and 58 were detected in the kernel-wash samples (**Table 3**). For the maize kernels stored in Kenya, 34 genera were identified from the UNITE database (**Table 4**). In the United States and Kenya, 31 and 16%, respectively, of the OTUs (37 United States and 26 Kenya) were not located in the UNITE database, which we refer to as not-assigned OTUs. A BLASTn analysis of these sequences to the NCBI database identified eight genera in the United States samples, which included five genera which were previously assigned from the UNITE database with the original set of OTUs (**Table 5**). A similar *BLASTn* analysis of the Kenya sequences identified six new genera two of which were not identified with the UNITE database. New genera not represented by the UNITE database analysis included *Epicoccum, Parastagnospora, Saccharomyopsis, Termitomyces*, and *Thielaviopsis* (**Table 5**).

**Table 3:** Genera identified from OTUs in maize kernels stored in Indiana (IN) and Arkansas (AR) after 3 months^a^.

Genus	Phyla	Category^b^	OTUs	Seqs	Initial^c^	IN PICS	IN Woven	AR PICS	AR Woven
*Alternaria*	Ascomycota	Field	7	83,347	+/+	+/+	+/+	+/+	+/+
*Bipolaris*	Ascomycota	Field	1	275	-/+	+/+	+/+	+/+	+/+
*Cephalosporium*	Ascomycota	Field	1	708	+/+	+/+	+/+	+/+	+/-
*Cercospora*	Ascomycota	Field	1	303	+/+	+/+	+/+	+/+	+/+
*Colletotrichum*	Ascomycota	Field	3	3968	+/+	+/+	+/+	+/+	+/+
*Curvularia*	Ascomycota	Field	1	1084	+/+	+/+	+/+	+/+	+/+
*Fusarium*	Ascomycota	Field	24	505,802	+/+	+/+	+/+	+/+	+/+
*Nigrospora*	Ascomycota	Field	6	44,945	+/+	+/+	+/+	+/+	+/+
*Ophiosphaerella*	Ascomycota	Field	1	233	+/+	+/-	+/+	+/+	+/+
*Ramularia*	Ascomycota	Field	1	84	+/+	-/-	-/-	-/+	-/-
*Sarocladium*	Ascomycota	Field	5	40,554	+/+	+/+	+/+	+/+	+/+
*Stagonospora*	Ascomycota	Field	1	238	+/+	+/+	+/+	-/+	+/+
*Stenocarpella*	Ascomycota	Field	12	243,944	+/+	+/+	+/+	+/+	+/+
*Trichoderma*	Ascomycota	Field	3	1531	+/+	+/+	+/+	+/+	+/+
*Acremonium*	Ascomycota	Other	2	711	+/+	+/+	+/+	+/+	+/+
*Byssochlamys*	Ascomycota	Other	1	1029	+/+	+/+	+/+	+/+	+/+
*Calcarisporium*	Ascomycota	Other	1	629	+/+	+/+	+/+	+/+	+/+
*Cladosporium*	Ascomycota	Other	4	3172	+/+	+/+	+/+	+/+	+/+
*Clonostachys*	Ascomycota	Other	1	1448	+/+	+/+	+/+	+/+	+/-
*Didymella*	Ascomycota	Other	1	125	-/+	+/+	-/+	+/+	+/+
*Eucasphaeria*	Ascomycota	Other	1	206	+/+	+/+	+/+	+/+	+/-
*Fusicolla*	Ascomycota	Other	1	575	+/+	+/+	+/+	+/-	+/-
*Geosmithia*	Ascomycota	Other	1	182	-/+	+/+	-/-	+/+	+/-
*Gibellulopsis*	Ascomycota	Other	1	379	+/+	+/+	+/+	+/+	+/+
*Hansfordia*	Ascomycota	Other	1	155	-/+	+/+	+/+	+/+	+/-
*Microascus*	Ascomycota	Other	1	200	-/+	+/+	+/+	+/+	-/+
*Neoascochyta*	Ascomycota	Other	1	312	-/-	-/+	-/-	-/-	-/-
*Phialemoniopsis*	Ascomycota	Other	1	640	+/+	+/+	+/+	+/+	+/+
*Pithoascus*	Ascomycota	Other	1	121	+/+	+/-	-/+	-/+	+/+
*Plectosphaerella*	Ascomycota	Other	1	144	-/+	+/+	+/+	+/+	+/-
*Pseudeurotium*	Ascomycota	Other	1	410	-/+	-/+	+/-	-/-	-/-
*Pseudogymnoascus*	Ascomycota	Other	1	1153	+/+	+/+	+/-	+/+	+/+
*Thelebolus*	Ascomycota	Other	1	6990	-/+	-/+	-/-	-/+	-/+
*Thermoascus*	Ascomycota	Other	1	113	+/+	+/+	-/+	+/+	+/+
*Thermomyces*	Ascomycota	Other	1	2196	+/+	+/+	+/+	+/+	+/+
*Aspergillus*	Ascomycota	Storage	8	23,659	+/+	+/+	+/+	+/+	+/+
*Monascus*	Ascomycota	Storage	1	1724	+/+	-/+	+/+	+/-	+/+
*Penicillium*	Ascomycota	Storage	11	32,718	+/+	+/+	+/+	+/+	+/+
*Xerochrysium*	Ascomycota	Storage	3	6721	-/+	+/+	+/+	+/+	+/+
*Xeromyces*	Ascomycota	Storage	5	72,265	+/+	+/+	+/+	+/+	+/+
*Aureobasidium*	Ascomycota	Yeast Like	2	2590	+/+	+/+	+/+	+/+	+/+
*Blastobotrys*	Ascomycota	Yeast Like	1	111	-/-	-/-	-/-	-/-	+/-
*Candida*	Ascomycota	Yeast Like	6	5105	+/+	+/+	+/+	+/+	+/+
*Cyphellophora*	Ascomycota	Yeast Like	1	152	+/+	+/+	+/+	+/+	+/+
*Debaryomyces*	Ascomycota	Yeast Like	1	1165	+/+	+/+	+/+	+/+	+/+
*Kazachstania*	Ascomycota	Yeast Like	1	174	+/+	+/+	+/+	+/-	+/-
*Trichomonascus*	Ascomycota	Yeast Like	2	1147	+/+	+/+	+/+	+/+	+/+
*Wickerhamomyces*	Ascomycota	Yeast Like	1	8597	+/+	+/+	+/+	+/+	+/+
*Amanita*	Basidiomycota	Other	1	211	-/-	-/+	-/-	-/-	-/-
*Russula*	Basidiomycota	Other	3	11,662	+/+	+/+	+/+	+/+	+/+
*Wallemia*	Basidiomycota	Storage	10	6321	+/+	+/+	+/+	+/+	+/+
*Bullera*	Basidiomycota	Yeast Like	1	321	+/+	+/+	+/+	+/+	+/+
*Cutaneotrichosporon*	Basidiomycota	Yeast Like	1	126	+/+	+/+	+/-	+/+	+/-
*Cystofilobasidium*	Basidiomycota	Yeast Like	1	330	+/+	+/+	+/+	+/+	+/-
*Guehomyces*	Basidiomycota	Yeast Like	1	31,851	+/+	+/+	+/+	+/+	+/+
*Malassezia*	Basidiomycota	Yeast Like	2	497	-/+	+/+	+/+	+/+	+/+
*Naganishia*	Basidiomycota	Yeast Like	4	3552	-/+	+/+	+/+	+/+	+/+
*Papiliotrema*	Basidiomycota	Yeast Like	3	979	+/+	+/+	+/+	+/+	+/+
*Piskurozyma*	Basidiomycota	Yeast Like	2	594	+/+	+/+	+/+	+/+	+/+
*Rhodosporidiobolus*	Basidiomycota	Yeast Like	1	174	+/+	+/+	+/+	+/+	+/-
*Rhodotorula*	Basidiomycota	Yeast Like	1	1009	+/+	+/+	+/+	+/+	+/+
*Trichosporon*	Basidiomycota	Yeast Like	1	349	+/+	+/+	+/+	+/+	+/-
*Mucor*	Zygomycota	Storage	5	4242	-/+	+/+	-/+	-/+	-/+

^a^*Each representative OTU sequence was queried against the UNITE data base. ^*b*^Fungi were categorized as field or storage fungi by their tendency for growth in maize prior to harvest or during the storage of maize. Fungi that did not readily colonize maize were classified as Other fungi. ^*c*^(+) indicated the presence of the genus and (-) indicates the genus not found in the Ground/Kernel-Wash*.

**Table 4:** Genera identified from OTUs in Kenya grain after 3 months of storage.

Genus	Phyla	Category^a^	Seqs	OTUs	Intial^b^	PICS	Woven
Alternaria	Ascomycota	Field	37,802	5	27	29	27
Bipolaris	Ascomycota	Field	2852	3	10	7	8
Cercospora	Ascomycota	Field	1397	1	12	17	21
Diaporthe	Ascomycota	Field	582	1	6	3	4
Exserohilum	Ascomycota	Field	12,999	2	16	11	14
Fusarium	Ascomycota	Field	2,613,917	56	30	30	30
Nigrospora	Ascomycota	Field	125,068	12	29	29	29
Pestalotiopsis	Ascomycota	Field	369	1	9	3	6
Sarocladium	Ascomycota	Field	202,065	7	30	30	30
Stenocarpella	Ascomycota	Field	58,006	4	27	30	30
Trichoderma	Ascomycota	Field	2256	1	8	5	5
Trichothecium	Ascomycota	Field	622	1	9	7	8
Cladosporium	Ascomycota	Other	2062	1	15	12	22
Clonostachys	Ascomycota	Other	1995	1	1	0	4
Dothiorella	Ascomycota	Other	663	1	5	3	1
Hirsutella	Ascomycota	Other	821	1	28	2	1
Lasodiplodia	Ascomycota	Other	27,445	2	24	22	21
Macrophomina	Ascomycota	Other	7205	1	11	10	8
Neofusicoccum	Ascomycota	Other	4802	1	12	5	13
Sphaeropsis	Ascomycota	Other	2151	1	6	2	3
Thelebolus	Ascomycota	Other	644	1	11	0	1
Xylaria	Ascomycota	Other	1853	1	2	22	22
Aspergillus	Ascomycota	Storage	21,589	9	23	17	29
Penicillium	Ascomycota	Storage	23,998	4	30	30	30
Xeromyces	Ascomycota	Storage	388	1	15	0	1
Aureobasidium	Ascomycota	Yeast Like	3108	1	17	11	15
Candida	Ascomycota	Yeast Like	29,486	6	30	30	30
Debaryomyces	Ascomycota	Yeast Like	398	1	7	1	2
Wickerhamomyces	Ascomycota	Yeast Like	590	1	10	4	1
Sporisorium	Basidiomycota	Field	496	1	26	3	2
Russula	Basidiomycota	Other	4754	2	30	21	22
Wallemia	Basidiomycota	Storage	11,113	5	26	13	30
Guehomyces	Basidiomycota	Yeast Like	2850	1	18	0	0
Papiliotrema	Basidiomycota	Yeast Like	472	1	20	10	13

^a^*Fungi were categorized as field or storage fungi by their tendency for growth in maize prior to harvest or during the storage of maize. Fungi that did not readily colonize maize were classified as Other fungi. ^*b*^Number of bags in which genera was identified, 30 total bags were tested for each bag type*.

**Table 5:** Genera identified by BLASTn analysis of the NCBI database with not-assigned category of OTUs.

Kenya	United States
Cladosporium (10)^a^	Aspergillus (1)
Fusarium (10)	Cladosporium (6)
Sarocladium (1)	Epicoccum (7)
Stenocarpella (1)	Fusarium (2)
Termitomyces (1)	Geosmithia (1)
Thielaviopsis (1)	Parastagnospora (1)
	Saccharomycopsis (1)
	Xeromyces (1)

^a^*Numbers in parentheses indicate the number of OTUs*.

We compared DNA isolated from kernel-washes and ground kernels. Two-thirds of the genera in the kernel-wash samples were also found in the ground samples, totaling 37 genera that represented over 97% of the identified United States sequence reads. Of the 63 total genera identified, 29 were among the United States core OTUs and comprised 95.6% of the identified reads. Seven genera comprised over 1% of the identified sequence reads from the ground samples. These consisted of *Fusarium* (47.2%), *Stenocarpella* (28.3%), *Sarocladium* (4.1%), *Alternaria* (3.8%), *Nigrospora* (3.6%), *Xeromyces* (1.9%), and *Aspergillus* (1.3%). Interestingly, 12 genera comprised over 1% of the identified sequence reads in the kernel-wash samples. These consisted of *Fusarium* (24.0%), *Xeromyces* (10.5%), *Alternaria* (9.8%), *Guehomyces* (5.8%), *Penicillium* (5.7%), *Stenocarpella* (3.6%), *Nigrospora* (3.0%), *Aspergillus* (2.5%), *Russula* (2.0%), *Sarocladium* (1.5%), *Wickerhammomyces* (1.3%), and *Thelebolus* (1.3%).

The five genera unique to the ground samples (*Blastobotrys*, *Fusicolla*, *Geosmitha*, *Pithoascus*, and *Stenocarpella*) represented only 0.15% of the identified sequences. Sixteen genera were unique to the kernel-wash samples and comprised 5.0% of the identified reads. Overall, *Fusarium* and *Stenocarpella* comprised about 56% of the total reads from the United States maize. For Kenya, *Fusarium*, *Sarocladium, Candida*, and *Penicillium* were identified in all samples and accounted for 82.6% of the sequence reads. The most prominent category of fungi in both the United States and Kenya were field fungi, which comprised 69.3 and 88.4% of the total reads, respectively (**Tables 3** and **4**). In the United States and Kenya, storage fungi comprised 11.0 and 1.6% of the total reads, yeast-like fungi comprised 4.3 and 1.1%, and other fungi comprised 2.4 and 1.2% of the total reads. Twelve genera were unique to the Kenya maize, including five field fungi (*Diaporthe*, *Exserohilum*, *Pestalotiopsis*, *Sporisorium*, and *Trichothecium*), five plant pathogenic genera (*Dothiorella*, *Ladodiplodia*, *Macrophomina*, *Neofusicoccum*, and *Sphaeropsis*), which are not pathogenic on maize, the entomopathogenic genus *Hirsutella*, and the wood decay genus *Xylaria*. A total of 35 genera were unique to the United States maize. These consisted of three storage fungi (*Monascus*, *Mucor*, and *Xerochrysium*), four field fungi (*Cephalosporium*, *Colletotrichum*, *Curvularia*, and *Stagonospora*), 11 yeast-like fungi, and 17 other fungal genera, including the tree-pathogenic genus *Geosmithia*.

### Geographic Location and Storage Bag Type

In Indiana and Arkansas a total of 63 genera were identified, between 43 and 56 genera were identified in every bag. Of these, 29 were determined to be core genera associated with both storage locations. The genera not in the core comprised 4.4% of the total reads. For the kernel-wash and the ground kernel United States samples, OTUs were analyzed for significant locational effects. In the kernel-wash samples no significant differences were observed in alpha diversity within samples and beta diversity between the kernel-wash samples. When individual OTUs from each location were compared, nine OTUs had significantly more reads in Indiana than Arkansas. These OTUs were assigned to *Fusarium* (1), *Nigrospora* (1), and *Wallemia* (5), two of the OTUs were not able to be identified. Analysis of the ground maize samples revealed no significant locational effects for both the alpha diversity and beta diversity metrics. Comparison of the individual OTUs revealed that five OTUs (*Penicillium*, *Trichomonascus*, and 2 unidentified OTUs) were more abundant in Arkansas and one OTU (*Thermomyces*) was more abundant in Indiana.

An analysis of the two bag types revealed no significant differences in alpha diversity for the kernel-wash data. For beta diversity, a comparison of the Bray–Curtis values between bag types revealed significant differences, suggesting that the species composition of PICS and woven bags diverged significantly during storage. However, none of the phylogenetic based beta diversity values were significantly different between bag types, suggesting that the significant differences observed between bag types were within closely related taxa. Eighteen OTUs from the kernel-wash samples underwent significant change associated with bag type. Taxonomic assignment of these OTUs revealed they were assigned to eight genera. Of these, 4 OTUs contained more reads from the PICS bags than the woven bags, which included the genera *Cladosporium* (1 OTU), *Cystofilobasidium* (1 OTU), and *Penicillium* (2 OTUs). The other 14 OTUs each had significantly more reads from the woven bags, which included the genera *Aspergillus* (5 OTUs), *Monascus* (1 OTU), *Wallemia* (2 OTUs), *Xerochrysium* (2 OTU), *Xeromyces* (1 OTU), and 3 OTUs that could not be identified.

For ground maize samples, there was no significant difference in alpha diversity between bag types. For beta diversity, the unweighted Unifrac values were significantly different between bag types, but Bray–Curtis values were not, suggesting that the significant differences observed between the PICS and the woven bags after 3 months of storage were likely in phylogenetically distinct taxa. Furthermore, 26 OTUs in the ground maize samples which underwent significant changes associated with bag type. Taxonomic analysis of these OTUs revealed they were assigned to eight genera. Of these, 6 OTUs contained more reads from PICS bags than from woven bags. These included the genera *Cercospora* (1 OTU), *Debaromyces* (1 OTU), *Sarocladium* (1 OTU), and 3 OTUs that could not be identified. The other 20 OTUs contained more reads from the woven bags, which included *Aspergillus* (5 OTUs), *Monascus* (1 OTU), *Wallemia* (7 OTUs), *Xerochrysium* (2 OTUs), *Xeromyces* (2 OTUs), and 3 OTUs that could not be identified.

In contrast to the United States grain, fewer genera were identified in the Kenya samples, with a median of 24 genera in each bag (range: 15–29). A comparison of the bag types from each of the 30 farms revealed an average of 19, 14, and 16 genera in the initial grain, PICS bags, and woven bags, respectively. From these 5, 5, and 6 genera were determined to be core within the initial, PICS, and woven bags, respectively. These cores represented approximately 84% of the total reads, and *Fusarium* comprised 89.7% of the core reads. Four genera were core in all three bags, *Fusarium*, *Candida*, *Penicillium*, and *Sarocladium*. The genus *Stenocarpella* was core to both treatments after storage, however, it was only detected in 27 of the 30 samples prior to storage. The genus *Wallemia* was found to be core to the woven bags and was identified in 26 of the initial samples, but was found in only 13 of the PICS bags. Interestingly, the ectomycorrhizal Basidiomycete, *Russula*, was found to be a core genera in the initial grain. After 3 months of storage the number of reads decreased 10-fold in both PICS and woven bags and was found in only 21 and 22 of the samples, respectively.

An analysis of alpha diversity metrics revealed that diversity was significantly lower within the initial Kenya samples than in PICS and woven bags after 3 months of storage (all metrics), even though the initial samples contained more genera. These results show that there were significantly fewer OTUs detected in the initial grain than after storage. The average number of observed OTUs per bag increased from 74 in the initial samples to 98 and 103 in the PICS and woven bags, respectively. Further, the significant increase of phylogenetic alpha diversity metrics during storage suggests that the majority reads from the initial samples were contained within a small number of closely related OTUs. In the initial grain, the most abundant OTU (*Fusarium*) comprised 69.5% of the total reads. After 3 months of storage this OTU decreased in relative abundance by 5.7 and 8.5% in the PICS and woven bags, respectively. This OTU was the most abundant OTU in all bags. In the initial grain 90% of the total reads were contained within the nine most abundant OTUs. These OTUs were assigned to the genera *Fusarium* (2 OTUs), *Sarocladium* (1 OTU), *Nigrospora* (1 OTU), *Stenocarpella* (1 OTU), *Lasodiplodia* (1 OTU), *Candida* (1 OTU), and 2 OTUs that could not be identified. After 3 months of storage this increased to 13 and 16 OTUs in the PICS and woven bags, respectively. OTUs in the PICS bags were assigned to *Fusarium* (7 OTUs), *Sarocladium* (1 OTU), *Alternaria* (1 OTU), *Nigrospora* (1 OTU), *Stenocarpella* (1 OTU), and 1 OTU that could not be identified. OTUs in the woven bags were assigned to *Fusarium* (8 OTUs), *Sarocladium* (2 OTUs), *Nigrospora* (1 OTU), *Stenocarpella* (1 OTU), *Aspergillus* (1 OTU), *Lasodiplodia* (1 OTU), and 2 OTUs that could not be identified. Of the OTUs listed above, 7 OTUs were highly prevalent in all bag types. These include the genera *Fusarium* (2 OTUs), *Sarocladium* (1 OTU), *Nigrospora* (1 OTU), *Stenocarpella* (1 OTU), and 2 OTUs not able to be identified. Overall, although the total number of genera detected in the grain decreased after storage, the relative decrease of fungi prevalent in the initial grain and the putative growth of storage and other xerophillic fungi during the experiment resulted in the reads being more evenly distributed amongst the OTUs, which resulted in an increase in alpha diversity measures.

There was no significant difference in alpha diversity between the Kenya PICS and woven bags. A comparison of beta diversity revealed significant overall differences in fungal composition between the bags. A pairwise comparison of bag types revealed significant differences in all beta diversity metrics comparing the composition of the PICS and woven bags from the initial grain samples. However, although the weighted and unweighted unifrac comparisons were significantly different, the Bray-Curtis dissimilarity index was not significantly different between the PICS and woven bags. While unifrac comparisons incorporate phylogenetic information to determine significant changes in community composition, the Bray–Curtis dissimilarity index compares OTU composition. This suggests that although there were not a large number of significant differences in OTU composition, the differences detected were phylogenetically distinct. A pairwise comparison revealed 83 of the 164 OTUs underwent significant changes in the PICS bags from the initial grain samples. Of these, 53 OTUs had significantly more reads in the PICS bags than the initial samples, while 30 OTUs had significantly more reads in the initial samples. The OTU with the most reads that underwent significant change was assigned to the genus *Fusarium* and comprised two-thirds of the total reads in the initial grain and PICS bags. This OTU contained significantly more reads in initial grain than the PICS bags. Interestingly, although the number of reads assigned to *Fusarium* fell after storage in the PICS bags, 33 of the 41 OTUs assigned to *Fusarium* contained more reads in the PICS samples. This suggests that the single *Fusarium* OTU played a significant role in the bag dynamics. A pairwise comparison of the initial and woven bag samples revealed 92 OTUs that underwent significant changes during storage. Of these 61 OTUs contained more reads from the woven bags than the initial samples, while 31 OTUs had significantly more reads in the initial samples. In stark contrast to previous comparisons, a comparison on the PICS and woven bags revealed 12 OTUs that underwent significant changes during storage. Of these, 9 OTUs contained more reads from the woven bags than the PICS bags. These OTUs were comprised of *Aspergillus* (3 OTUs), *Bipolaris* (1 OTU), *Nigrospora* (1 OTU), and *Wallemia* (4 OTUs). The remaining 3 OTUs contained more reads from the PICS bags than the woven bags, these were assigned to the genus *Fusarium*.

## Discussion

Although over 150 species of yeast-like and filamentous fungi have been reported in stored grains (Meronuck, 1987), typical dilution plating methods often skew population analyses in favor of the most abundant species, whereas slow-growing, less abundant, or difficult to culture organisms are underrepresented. The use of HTS to profile the fungal microbiome of grains allows the identification of many previously unrecognized fungal associations. The maize used in this study was stored for 3 months in hermetic and woven bags in West Lafayette, IN and Marianna, AR (Lane and Woloshuk, 2017) and in Makueni County, Kenya (Maina et al., 2016, 2017). Although the study in Kenya focused on the presence of *Aspergillus* spp. and *Fusarium* spp., Lane and Woloshuk (2017) identified primarily species of *Gibberella*, *Aspergillus*, *Penicillium*, and *Alternaria* cultured from kernel-washes and from surface sterilized kernels. In addition, many fungal colonies were not identifiable based on morphological characteristics (Lane and Woloshuk, 2017). The bioinformatics analysis of 1,377,221 DNA sequences identified 59 additional genera not found in the plating study by Lane and Woloshuk (2017). Similarly, the analysis of 3,633,944 DNA sequences from maize kernels stored in PICS and woven bags in Makueni County, Kenya revealed 32 genera in addition to the *Aspergillus* and *Fusarium* spp. previously identified. Others have found similar results, including a study of the microflora on wheat heads in Australia by Barkat et al. (2016) who identified only 15 fungal genera from nearly 500 colonies isolated by plating techniques. The authors also used HTS on DNA isolated from bulked samples of wheat heads, which revealed at least three additional genera (*Paecilomyces*, *Cryptococcus*, and *Aspergillus*). A study by Yuan et al. (2018) also identified 81 genera from wheat kernels using HTS of the ITS2 region. An analysis HTS data by Ding et al. (2015) from stored peanuts in China found that *Alternaria*, *Aspergillus*, *Cladosorium*, *Clonostachys*, *Emericella*, *Eurotium*, *Penicillium*, and *Talaromyces* were the predominant fungi. In another study on stored peanuts, Xing et al. (2016) found that the predominant members of the fungal microbiome were *Aspergillus, Penicillium*, *Rhizopus*, and *Wallemia*, which are commonly identified by traditional plating (Bhattacharya and Raha, 2002; Nakai et al., 2008). Their HTS analysis also identified an additional 41 genera in the fungal microbiome (Xing et al., 2016). Our results revealed that similar to the previous wheat and peanut studies the major genera identified by traditional dilution plating methods and by plating surface sterilized kernels were also identified by HTS analysis.

The number of genera identified varied greatly between the United States and Kenya samples. Samples from the United States had a median of 51 genera identified from each bag (range: 43–56). In contrast, samples from Kenya had a median of only 24 genera (range: 15–29) from each bag, even though substantially more bags were analyzed. The difference could be attributed to the quality of the initial grain used in the United States study, which contained a large amount of grain dust. Previous studies have demonstrated the fungal diversity of grain dust. A study by Hill et al. (1984) identified over 14 genera of fungi in samples of grain dust collected from harvesters and grain elevators, including *Aspergillus*, *Gibberella*, and *Penicillium*. Martin and Sauer (1976) also found that the number of microorganisms in grain dust was higher than in the grain mass.

Different environments can create selection pressure for distinct fungal taxa to thrive. El-Kady and Youssef (1993), found that distinct fungal species dominated soybean samples obtained from two locations with distinct temperature differences, 28 and 45°C. Although distinct temperature differences between Indiana and Arkansas were observed in this United States study, an analysis of the diversity revealed no overall significant changes between grain stored in Indiana and Arkansas. However, analysis revealed several individual OTUs underwent significant changes in relative abundance. Taxonomic assignment of these OTUs indicated they include the storage fungi *Wallemia*, which grows under a variety of conditions but is most active at 20°C (Wheeler et al., 1988); similar conditions were observed in Indiana where *Wallemia* was most prevalent.

Other studies have indicated that the fungal microbiome of stored grain is dependent on the location harvested. A study of the fungal microbiome of barley grain in Western Canada by Chen et al. (2016) in six distinct environments found differences in fungal diversity among the locations with less than 2% of OTUs being common among the locations. Unlike this Canadian study, our initial grain source for storage in Indiana and Arkansas had been stored from the fall to the start of the experiment in July. Despite changes in relative fungal populations between Indiana and Arkansas, few changes were observed in the number of OTUs detected between these locations; 95% of the OTUs were common between Indiana and Arkansas. Thus, the fungal microbiome may have been established, and the lack of significant change in OTUS (*P* > 0.05) after 3 months of storage may reflect the poor ingression of fungal species that are adapted to the higher temperatures in Arkansas.

Unlike the maize kernels stored in the United States, the Kenya grain was not obtained from a single source. Similar to the results of Chen et al. (2016), our results indicate a significant amount of diversity in OTUs between farm locations in Kenya. Samples taken from initial grain samples revealed an average of 74 OTUs per farm, of which only 17 were common among all farms. In contrast to samples stored in the United States, the fungal microbiome of the grain stored in Kenya changed significantly during storage. After 3 months, the average number of OTUs per farm increased to 98 and 103 in the PICS and woven bags, respectively. In each bag, approximately 50% of the OTUs had been detected in the initial samples, indicating the growth of previously undetected fungi during storage and establishment of the post-harvest microbiome. Perhaps, grain drying inhibited the growth of the field fungi that were identified in the initial samples and that, during storage, xerophillic fungi were at a competitive advantage (Christensen and Kaufmann, 1965).

In the United States study, isolating DNA from maize kernels by kernel-washes and whole-kernel grinds produced starkly different results in fungal microbiome composition. Previous studies have demonstrated the difference in the microbiome of the grain surface and within grain. A study by Hill and Lacey (1983) demonstrated the prevalence of yeast-like fungi on the surface of ripening barley as well as the leaf pathogen *Alternaria alternata*. A study by Xing et al. (2018) analyzed the mycobiota of maize kernels stored at room temperature between 6 months and 12 years. Fungi were isolated from both the seed surface and within the kernel using similar methodology to our study. The authors identified 16 fungal species, two of which were unique to the seed surface and seven of which were isolated only from within the maize kernels. In the United States study, the most noticeable difference was the lower relative abundance of field fungi in kernel-wash samples (42.4% of kernel-wash reads) compared to the ground samples (87.6% of ground reads). The two most prevalent field fungi in the United States samples were *Fusarium* and *Stenocarpella*. With the moisture content of the maize in the bags below 16%, neither *Fusarium* nor *Stenocarpella* species can grow because the minimum moisture content for growth of *Fusarium graminearum* and *Stenocarpella maydis* is 23.8% (Koehler, 1959). Under low moisture contents, these fungi become quiescent (Pereyra et al., 2004), supporting their presence primarily in the ground samples. In contrast, the other categories of fungi were present in higher relative abundance in the kernel-wash reads. In the ground maize samples, storage fungi and yeast-like fungi comprised 4.0% and 0.8% of the total reads, respectively, while in the kernel-wash samples these reads comprised 20.1% and 9.6% of the total reads. After initial infection of the grain, storage fungi sporulate on the surface of the grain and produce a large amount of conidia on the surface (Payne, 1998). Yeast-like fungi grow epiphytically on the surface of plants and thus are prominent in the kernel-washes. A study by Di Menna (1959) analyzed the epiphytic yeast flora of pasture grass in New Zealand and determined that approximately 1% of the wet mass of the leaf is comprised of yeast-like fungi. It is also likely that the significant decrease in the relative abundance of field fungi on the surface of the grain during storage caused a significant increase in the relative abundance of the storage and yeast-like fungi, although some growth of storage and yeast-like fungi was likely.

Hermetic bags are impermeable to gasses and moisture, whereas woven bags allow the exchange of oxygen, carbon dioxide, and moisture (Murdock and Baoua, 2014). In the United States study, fungal colony counts and the percentage of infected kernels increased significantly in the grain stored in woven bags, whereas no significant increase was found in the hermetic bags (Lane and Woloshuk, 2017). The fungal microbiome results indicated that the field fungi, *Fusarium* and *Stenocarpella*, dominated the reads in the HTS data. For both bag-types, the relative abundance of *Fusarium* species fell significantly during storage, from an initial 55.7% of the total reads to 36.0% and 30.0% of the total reads in the hermetic and woven bags, respectively. These results are consistent with those of Magan and Lacey (1984a) who reported that storage fungi outcompete field fungi under lower moisture storage conditions. A study by Xing et al. (2017) explored the distribution of mycotoxigenic fungi in maize before drying (average moisture content 22.14%) and after 2 months of storage by farmers (average moisture content 12.13%). Similar to the maize stored in our study, *Fusarium* was the most common fungus identified prior to storage. The authors also observed that the prevalence of most fungi within the grain mass fell during 2 months of storage. In our study, the prevalence of the field fungi, *Fusarium* fell during storage. Similarly, in Xing et al. (2017) the prevalence of *Fusarium verticillioides*, and *Fusarium* sp. also fell during storage. However, *F. graminearum* increased during storage. The authors hypothesize this is likely due to the rapid growth of *F. graminearum* during the drying process. In our study, the grain was dried prior to the initial sampling and the effect of the drying process on the microbiome was not determined. In the Kenya grain, *Fusarium* dominated the reads in the sequence data; however, little change was seen in the relative abundance of the *Fusarium* reads between bag types, from an initial 74.2% of the total reads to 77.7% and 74.1% of the total reads in the PICS and woven bags, respectively. Although Magan and Lacey (1984a) demonstrated that storage fungi are antagonistic to field fungi, the storage fungi were not yet prominent in the grain microbiome at the start of the Maina et al. (2016, 2017) experiment, possibly limiting the antagonistic effects of the storage fungi.

Fungal microbiome results indicated the presence of several xerophillic filamentous fungi, including, *Aspergillus*, *Monascus*, *Mucor, Penicillium*, *Wallemia*, *Xerochrysium*, and *Xeromyces*. With the exception of *Mucor* and *Penicillium*, these genera were present primarily in the woven bags in both storage locations in the United States study. Although *Mucor* contained only 0.3% of the total reads, these reads were found primarily in the hermetic bags. Members of the genus *Mucor* are dimorphic and can grow as filamentous hyphae or in a yeast-like form. A study by Bartnicki-Garcia and Nickerson (1962) demonstrated that increased atmospheric carbon dioxide levels induced yeast-like development of *Mucor* and anaerobic growth. Hermetic bags create a low-oxygen, high-carbon dioxide environment which is conducive to the growth of *Mucor* (Murdock and Baoua, 2014). *Penicillium* was also found primarily in the hermetic bags. It was not possible to discern species of *Penicillium.* However, several species of *Penicillium*, such as *P*. *hordei*, *P*. *roquefortii*, and *P*. *verrucosum*, can grow in low oxygen environments (Magan and Lacey, 1984a,b; Petersson and Schnürer, 1999). The genera *Wallemia* and *Xerochrysium* were found primarily in the woven bags in Indiana and Arkansas, respectively. The most notable increase during storage was in the storage fungi *Xeromyces* which increased from 0.05% of the initial reads to 22.3% of the reads in the woven bags in Arkansas while comprising only 4.0% of the reads in the woven bags in Indiana. A study by Leong et al. (2011) demonstrated that the storage fungi *Xeromyces bisporus* experiences optimal growth at 30°C, a temperature observed primarily in Arkansas. Further, this study demonstrated the competitive growth of *X. bisporus* against other storage fungi at low water activity.

The four xerophillic genera, *Aspergillus, Penicillium, Wallemia*, and *Xeromyces* were detected in the maize from the Kenya study. Despite the presence of only four genera, the Kenya samples contained a higher relative abundance of storage fungi prior to storage than the samples from the United States study. However, the storage fungi were more established in the United States grain because of the poor quality of the grain and the higher initial level of fungi detected. Results from the initial samples indicated the presence of *Penicillium* reads, agreeing with Maina et al. (2016, 2017) who also indicated the presence of *Penicillium* in the initial samples. Although primarily a storage fungus, previous studies have documented the ability of *Penicillium* to colonize maize under field conditions (Koehler, 1959; Mukanga et al., 2010). However, in contrast to the United States study, the abundance of *Penicillium* fell during storage in the hermetic bags in Kenya. During storage, the overall abundance of storage fungi in the woven bags rose from an initial 1.5 to 3.0%. The majority of these increases were observed in the genera *Aspergillus* (1.5% increase) and *Wallemia* (0.8% increase) in the woven bags.

Multiple studies have demonstrated that when grain moisture is high, yeast-like fungi proliferate on the grain surface under hermetic conditions (Weinberg et al., 2008; Williams et al., 2014; Tubbs et al., 2016). In this study, the initial grain moisture was 14.0% (Lane and Woloshuk, 2017). After 3 months of storage, the moisture content was 14.2 and 14.3% in Indiana and Arkansas, respectively. Despite the low moisture content, the overall relative abundance of yeast-like fungi rose from 2.4 to 4.8% of the overall reads. These fungi underwent their largest change in the hermetic bags where their relative abundance rose from the initial 2.3 to 4.0% and 10.8% in Indiana and Arkansas, respectively. Among these fungi, *Guehomyces* increased in the Arkansas PICS bags from 0.1% of the initial reads to 19.7% of the reads after 3 months of storage. Members of this yeast-like fungal genus are associated with fermentation (Batra and Millner, 1974; Aidoo et al., 2006).

In summary, our study has demonstrated that the fungal microbiome associated with stored maize contains a diverse population of fungi. The sequence of the ITS2 region, which is commonly used in fungal microbiome studies (Ding et al., 2015; Chen et al., 2016; Hertz et al., 2016; Liebe et al., 2016; Xing et al., 2016; Yuan et al., 2018), yielded 310 OTUs that could be matched to sequences in the UNITE database and assigned to 75 genera. The fungi in the United States study that were identified by dilution plating (Lane and Woloshuk, 2017) were also among the predominant genera found in the fungal microbiome sequence data. In addition, we discovered that *Stenocarpella*, which does not readily produce conidia in culture, was also among the predominant genera. *S. maydis* is a common ear rot pathogen in Indiana, which makes its presence not surprising. Several OTUs underwent significant changes during storage in the hermetic PICS and non-hermetic woven bags. The results also indicate that while storage bag type played a significant role in both the United States and Kenya, storage location did not appear to have a significant effect on the microbiome of grain stored in the United States. Overall, the results suggest that the majority of genera in the fungal microbiome were fixed in the initial grain, and the storage treatment had only a small impact on the overall diversity of genera. In contrast, the grain used in the Kenya study was stored directly after harvest and drying. Thus, the microbiome of the grain used in this study was not established before the start of the experiment and consequently more diverse after storage. This study also determined that kernel washes provide a profile of the fungi on the surface of the fungi. However, DNA extracts from the entire kernel captured most of the surface fungi as well as those within the grain. Finally, these results concur with the previous literature and indicate that the use of hermetic storage bags helps to maintain the integrity of the grain and its microbiome. Although the proper drying of the grain mass remains essential for safe storage, these results indicate that the use of hermetic bags can mitigate many risks associated with grain storage, including the growth of mycotoxigenic fungi. The results also indicate that hermetic storage remains effective over a wide variety of environmental conditions. However, the results also demonstrate that the microbiome of the grain mass during storage is dependent on the microbiome at commencement of storage. Thus, to ensure the quality of the grain mass during storage, farmers should apply good management practices during grain harvest and drying.

## Author Contributions

BL, AM, BB, JW, and CW were responsible for conception and design of the study. AM and JW were responsible for collection of Kenya experiment. BL, SS, and BB were responsible for the United States experiment. BL, SS, CN, AM were responsible for processing samples and analysis of data. BL and CW wrote the draft manuscript and all authors contributed to the final version.

## Conflict of Interest Statement

The authors declare that the research was conducted in the absence of any commercial or financial relationships that could be construed as a potential conflict of interest.
